# Construction of a prognostic 6-gene signature for breast cancer based on multi-omics and single-cell data

**DOI:** 10.3389/fonc.2023.1186858

**Published:** 2023-11-21

**Authors:** Zeyu Xing, Dongcai Lin, Yuting Hong, Zihuan Ma, Hongnan Jiang, Ye Lu, Jiale Sun, Jiarui Song, Li Xie, Man Yang, Xintong Xie, Tianyu Wang, Hong Zhou, Xiaoqi Chen, Xiang Wang, Jidong Gao

**Affiliations:** ^1^ National Cancer Center, National Clinical Research Center for Cancer, Cancer Hospital, Chinese Academy of Medical Sciences and Peking Union Medical College, Beijing, China; ^2^ National Cancer Center, National Clinical Research Center for Cancer, Cancer Hospital & Shenzhen Hospital, Chinese Academy of Medical Sciences and Peking Union Medical College, Shenzhen, China; ^3^ Department of Scientific Research Projects, Beijing ChosenMed Clinical Laboratory Co. Ltd., Beijing, China

**Keywords:** breast cancer, biomarker, multi-omics, risk score, prognosis, triple-negative breast cancer

## Abstract

**Background:**

Breast cancer (BC) is one of the females’ most common malignant tumors there are large individual differences in its prognosis. We intended to uncover novel useful genetic biomarkers and a risk signature for BC to aid determining clinical strategies.

**Methods:**

A combined significance (*p*_combined_) was calculated for each gene by Fisher’s method based on the RNA-seq, CNV, and DNA methylation data from TCGA-BRCA. Genes with a *p*_combined_< 0.01 were subjected to univariate cox and Lasso regression, whereby an RS signature was established. The predicted performance of the RS signature would be assessed in GSE7390 and GSE20685, and emphatically analyzed in triple-negative breast cancer (TNBC) patients, while the expression of immune checkpoints and drug sensitivity were also examined. GSE176078, a single-cell dataset, was used to validate the differences in cellular composition in tumors between TNBC patients with different RS.

**Results:**

The RS signature consisted of *C15orf52*, *C1orf228*, *CEL*, *FUZ*, *PAK6*, and *SIRPG* showed good performance. It could distinguish the prognosis of patients well, even stratified by disease stages or subtypes and also showed a stronger predictive ability than traditional clinical indicators. The down-regulated expressions of many immune checkpoints, while the decreased sensitivity of many antitumor drugs was observed in TNBC patients with higher RS. The overall cells and lymphocytes composition differed between patients with different RS, which could facilitate a more personalized treatment.

**Conclusion:**

The six genes RS signature established based on multi-omics data exhibited well performance in predicting the prognosis of BC patients, regardless of disease stages or subtypes. Contributing to a more personalized treatment, our signature might benefit the outcome of BC patients.

## 1 Introduction

Breast cancer (BC) is one of the females’ most common malignant tumors (1). Although BC with no metastasis was considered a curable disease, due to the enormous cardinal number and lack of advanced diagnosis and therapy, some patients could not be diagnosed at an early stage (1, 2). At the same time, the early detection rate of BC in China is less than 20%, and the 5-year survival period is slightly lower than that in western developed countries (3). Some molecular subtypes of BC, such as triple-negative breast cancer (TNBC), have a poor prognosis (4). Finding valuable markers to distinguish the prognosis of BC can improve the efficiency of clinical diagnosis and treatment, reducing treatment-related toxicities and thus reducing the occurrence of adverse health outcomes. Therefore, finding biomarkers that can effectively predict the prognosis of BC is of great significance.

Compared with normal tissue, various abnormal genetic changes occurred in the tumor tissue, manifesting as abnormal gene structure and function. Traditional single-omics studies are complex to fully reveal gene changes in the tumor tissue while using multi-omics data provides an opportunity to uncover deeper insights (5, 6). Several recent studies have demonstrated that multi-omics data identified novel biomarkers from new perspectives, which can improve cancer patients’ diagnosis, treatment, and prognosis (7–10).

Although there have been many previous studies on breast cancer biomarkers, they all have limitations. Shen et al. used a prognostic signature consisting of 11 lncRNA associated with immune cell infiltration to effectively predict the prognosis of patients with breast cancer (11); however, the study used 11 lncRNA, which increased the cost of the clinical study and did not use an additional validation data set to validate the prognostic signature. Chen et al. used a prognostic signature consisting of 16 pyroptosis-related genes to predict the prognosis of breast cancer patients (12); Liu et al. screened by various methods to obtain a biomarker consisting of 7 lncRNA that could effectively predict the prognosis of patients and found to be related to the immune infiltration of patients (13). However, these studies used more genes to form the prognostic signature than the present study to achieve the ability to effectively predict the prognosis of patients, resulting in a more costly and less applicable clinical study. In contrast, only RNA expression data were used to screen hub genes, resulting in the lack of robustness of the results obtained from the screening. In this study, the prognostic signature used fewer genes and a combination of three data dimensions to screen for hub genes, resulting in a more reliable prognostic signature.

Fisher’s method is the most broadly applied p-value combination tests method, which can integrate information from multiple omics into one feature (14, 15). In the multi-omics study based on a cancer sequencing database, such as The Cancer Genome Atlas (TCGA), we have different dimensions of information for the same genes, such as RNA sequencing (RNA-seq), copy number variations (CNV), and DNA methylation data, and tests for each dimension of data offer distinct characteristics of the marker genotype (16, 17). With the combination of the p-value for the tests, we could screen gene markers associated with BC from multiple aspects.

Tumor immunity is another important factor affecting the prognosis of tumor patients. Abundant immune cells infiltrate the tumor microenvironment (TME), called tumor-infiltrating immune cells, which were considered to perform a bidirectional role in tumor development in distinct cancers (18). Immune checkpoints are a series of components expressed in TME, believed to affect the antitumor response of T-cells (19). Elucidating the effect of genetic markers for prognosis on tumor-infiltrating immune cells and immune checkpoints might benefit the treatment and survival of BC patients (20, 21).

In this study, we aimed to identify several genes associated with the prognosis of all subtypes of BC patients that could become potential biomarkers in the multi-omics data from The Cancer Genome Atlas (TCGA) database and form a risk score (RS) signature. The results indicated that the RS signature developed in this study could effectively predict the prognosis of BC patients with higher predictive power than traditional clinical indicators, and was applicable to all subtypes of BC.

## 2 Methods

**Figure 1:**
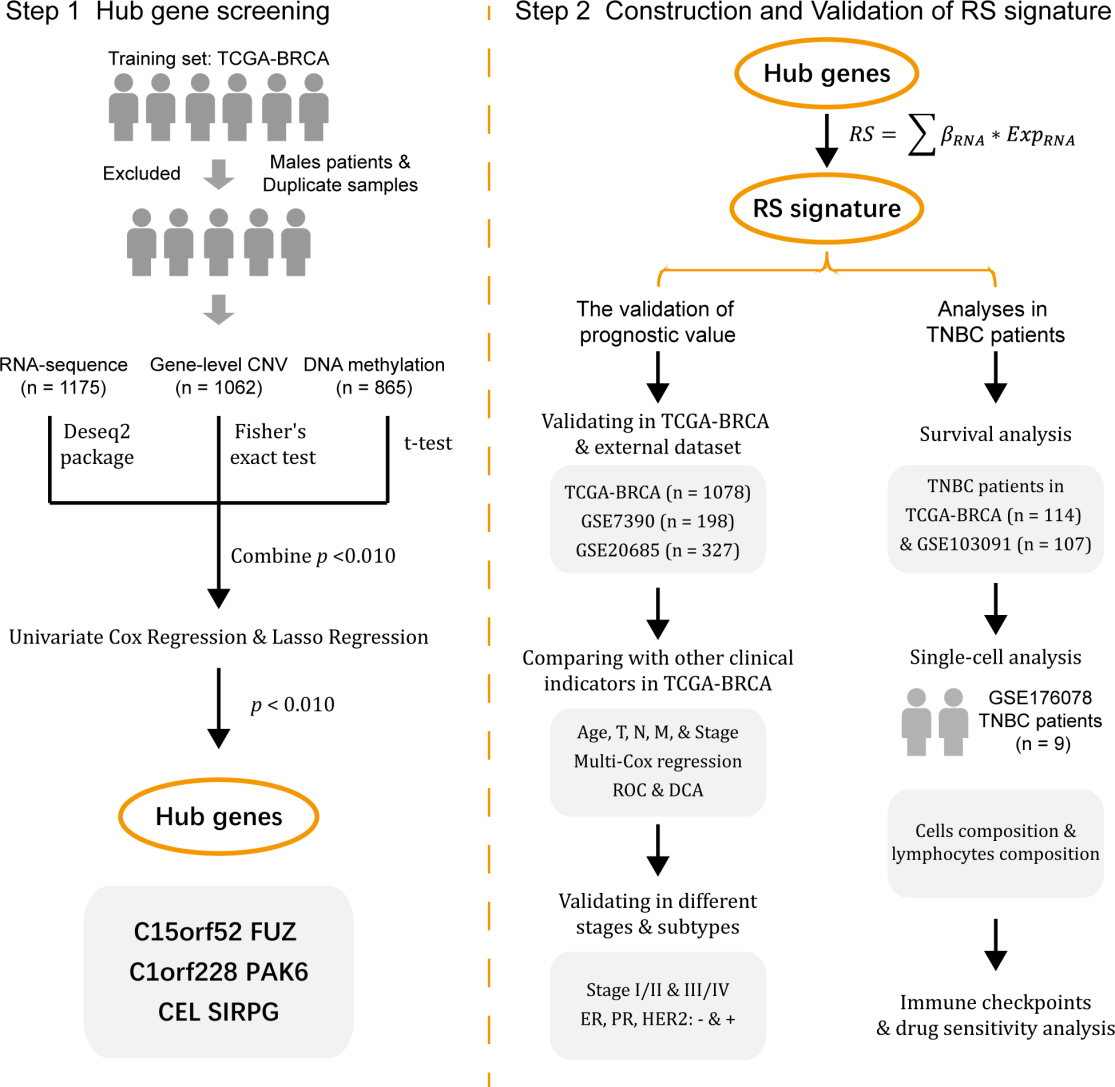
Workflow of this study.

The workflow of the study is shown in **Figure 1**.

### 2.1 Data preparation and processing

The RNA sequencing (RNA-seq) level-3 gene expression data (n = 1217), GISTIC2 method estimated gene-level CNV data (n = 1104), DNA methylation 450k array data (n = 890), as well as clinical (n = 1284) and survival information (n = 1260) of BC patients in TCGA were obtained from the University of California Santa Cruz (UCSC) database (https://xenabrowser.net/datapages/). While the RNA-seq data and related clinical and survival information of GSE7390 (n = 198), GSE20685 (n = 327), and GSE103091 (n = 107) were obtained from Gene Expression Omnibus (GEO) database (http://www.ncbi.nlm.nih.gov/geo/).

As the imbalances due to sex chromosomes in methylation analysis and the heterogeneity of male BC (22–24), we excluded all male patients in the TCGA dataset before data analysis. At the same time, a small number of duplicated samples from the same patients were also excluded (in the TCGA database, 01-09 represents tumor samples, and 10-19 represents normal samples, while sometimes one type of sample from the same patient will appear more than once). Finally, 1175, 1062, and 865 samples from the RNA-seq, gene-level CNV, and DNA methylation 450k array data of the TCGA database were included.

The TCGA datasets were used as the training set, and GSE7390 and GSE20685 were used as validation sets 1 and 2. Since the TNBC subtype was considered to have the worst prognosis in breast cancer, we chose GSE103091 as an external validation set for the analysis in TNBC patients.

### 2.2 Hub genes screening

First, only genes present in all three datasets (RNA-seq, gene-level CNV, and DNA methylation 450K) were retained in the training set (15649 overlapped genes in total). The differences in RNA expression between tumor and normal tissues were determined by the DESeq2 R package (version 1.34.0) (25), while Fisher’s exact test determined the differences in gene-level CNV and DNA methylation and t-test, respectively. After getting three independent p-values, namely *p*_RNA_, *p*_CNV_, and *p*_MET_, for each gene from the three omics, we calculated the combined p-value (*p*_combined_) using Fisher’s method, and the calculation process of *p*_combined_ was shown in equation (1). In this equation, pi represents *p*_RNA_, *p*_CNV_, and *p*_MET_, and S is a statistic.


(1) $$\text{S}=\;-2\log_{e}\prod_{i}p_{i}\;$$

In Fisher’s method, the statistic S was first calculated by *p*_RNA_, *p*_CNV_, and *p*_MET_, and the statistic S was transferred into the *p*_combined_ based on a chi-square distribution with 2k degrees of freedom (k = 3 in the present study).

Then genes with *p*_combined_< 0.010 were screened out, and univariate cox regression analysis with overall survival (OS) was performed based on the RNA-seq and DNA methylation datasets. Genes with *p*-value< 0.010 in both datasets were considered significant, and then Lasso regression analysis in RNA-seq and DNA methylation datasets were conducted. Genes that showed significant association with OS in Lasso regression in both RNA-seq and DNA methylation datasets would be treated as hub genes which were considered to be highly correlated with the prognosis of BC patients.

### 2.3 Construction and validation of RS signature

To construct an RS signature relying on the hub genes selected above, we first calculated the risk score of each patient in the training set with equation (2). In this equation, β_RNA_ represents the coefficient in the univariate cox regression analysis of the hub genes, and Exp_RNA_ represents the expression of the hub genes in the RNA-seq data.


(2) $$Risk\;score=\;\sum^{}\beta_{RNA}*Exp_{RNA}\;$$

Then the BC patients in the training set were divided into two subgroups, high-risk and low-risk, with the median RS of all patients as the cut-off value. The Kaplan-Meier (KM) method was used to compare the difference in OS between the two subgroups and form a survival curve, while a receiver operating characteristic (ROC) curve was drawn, and the area under the ROC curve (AUC) was considered to indicate the accuracy of RS signature in predicting the prognosis of BC patients.

After that, similar analyses were conducted in validation sets 1 and 2 to assess the predictive accuracy of the RS signature.

### 2.4 Evaluation of the predictive value of RS signature

Other analyses were conducted to evaluate the prognostic value of the RS signature established in the training set. First, the predictive ability for prognosis in BC patients from the training set of the RS signature was compared with other traditional clinical factors, including age, tumor topology (T), regional lymph node (N), metastasis (M), and American Joint Committee on Cancer (AJCC) TNM stage. Multivariate cox regression was used to test the independent predictive ability of the RS signature and a Nomogram was drawn to visualize better the impact of RS and other clinical indicators on the prognosis of patients. At the same time, the ROC curves of RS and other clinical factors were drawn to compare the predictive power between different prognostic factors with AUC as evaluating indicator. To better evaluate the value of RS in clinical decision-making, we performed decision curve analysis (DCA), while the area under decision curves (AUDC) was used to assess the value of RS and other clinical indicators.

Second, to evaluate the prognostic value of RS signature in the patients with different TNM stages and molecular subtypes, patients in the training set were stratified by their TNM stages or molecular subtypes, in which KM and ROC analyzes were conducted. In this process, patients in the training set were divided into two groups based on their TNM stage (stage I or II and stage III or IV). While the clinical information of the training set contains the immunohistochemical results for estrogen receptor (ER), progesterone receptor (PR), and human epidermal growth factor receptor 2 (*HER2)* expression in tumor samples, patients were divided into ER-negative or -positive, PR-negative or -positive, and *HER2*-negative or -positive, respectively. For each group of patients, survival and ROC curves were drawn.

Finally, the predictive performance of RS in TNBC was additionally analyzed as TNBC was considered to have a worse prognosis (26). A Violin plot was first performed to illustrate the distribution of RS among TNBC and non-triple-negative BC (NTNBC) patients in the training set. Then KM and ROC analyses were conducted on the TNBC patients from the training set. While an external validation set, GSE103091, containing 107 TNBC patients, was introduced to evaluate the RS signature’s performance in predicting TNBC patients’ prognosis.

### 2.5 Single-cell data preparation and analysis

The single-cell RNA sequencing (scRNA-seq) data and matched bulk RNA-seq data of GSE176078 were obtained from the GEO database (http://www.ncbi.nlm.nih.gov/geo/). GSE176078 contained scRNA-seq data from 26 BC patients (including 11 ER-positive, 5 *HER2*-positive, and 10 TNBC, a total of 130,246 single cells are included), 24 of which have matched bulk RNA-seq data. After screening, we included 9 TNBC patients with scRNA-seq and bulk RNA-seq data for further analysis.

The Seurat R package (version 4.3.0) (27) was applied to analyze scRNA-seq data. After data normalization, we used the FindClusters function to cluster the cells data from the 9 TNBC patients (resolution = 0.3), while UMAP reduction of cell clustering was also performed. Meanwhile, the SingleR R package (version 2.0.0) (28) was used to identify cell types, and the RS of TNBC patients were calculated using the matched bulk RNA-seq data. The lymphocyte subpopulations in the cells were further screened by obtaining differences in the cellular composition of different RS patients, and the differences in the composition of lymphocytes in the tumors were further analyzed, as well as the expression of the screened genes in the different cell types was demonstrated.

### 2.6 Immune checkpoint analysis

The expression of immune checkpoints first went through logarithmic transformation (base = 10), then the expression of 33 known immune checkpoints were examined. Boxplot was used to illustrate the expression of immune checkpoints among high-risk and low-risk subgroups. Wilcoxon rank-sum test was used to evaluate whether there were differences in the distribution of immune checkpoints expression between high-risk and low-risk TNBC patients, and the p-values were adjusted using the FDR method.

### 2.7 Drug sensitivity analysis

In the drug sensitivity analysis, the data from The Genomics of Drug Sensitivity in Cancer (GDSC) database was used as a training matrix. The half maximal inhibitory concentration (IC50) was calculated by oncoPredict (version 0.2) R package basing on the training matrix to derive the difference in drug sensitivity between high-risk and low-risk subgroups of TNBC patients.

### 2.8 Statistical methods

All data analysis was done by R (version 4.2.1). Survival analysis was conducted with the survival package (version 3.3.1), and forest plots were drawn by the forestplot package (version 2.0.1). Further KM and ROC analyses were conducted with the survminer (version 0.4.9) and timeROC (version 0.4) (29) packages, while the corresponding figures were also drawn with the same packages. DCA was performed using ggDCA package (version 1.2). Heatmap was drawn by the pheatmap package (version 1.0.12), Venn diagram was drawn by the venn package (version 1.11), and other figures were drawn by the ggplot2 (version 3.3.6) and ggpubr (version 0.4.0) packages. In the violin plots, the Wilcoxon rank-sum test was used to evaluate whether the difference between groups was statistically significant.

All statistical tests are two-sided, and in most analyses, p ≤ 0.050 were considered statistically significant except for the gene screening step, in which p ≤ 0.010 is the screening criterion.

## 3 Results

### 3.1 Identify hub genes with multi-omics data in the training set

In the BC training dataset, most of the 15,649 overlapped genes were altered in at least one dimension. Only 153 genes had a p_combine_ > 0.010 and were excluded in the subsequent analysis. After univariate cox regression, 592 genes from RNA-seq data and 1891 genes from DNA methylation data showed significant association with OS. There were 83 overlapped genes in the two gene sets, and Lasso regression analysis was performed on these 83 genes. Then 29 and 14 genes were selected by Lasso regression in RNA-seq and DNA methylation data, respectively. Then six overlapped genes shared by both Lasso regression of RNA-seq and DNA methylation data, namely *C15orf52*, *C1orf228*, *CEL*, *FUZ*, *PAK6*, and *SIRPG*, would serve as hub genes. The Venn diagrams of the screening process of genes are shown in **Figures S1A, S1B**, and the expression of the six genes in tumor and normal tissue is shown in **Figure S1C**.

### 3.2 Construction and validation of the RS signature

**Figure 2:**
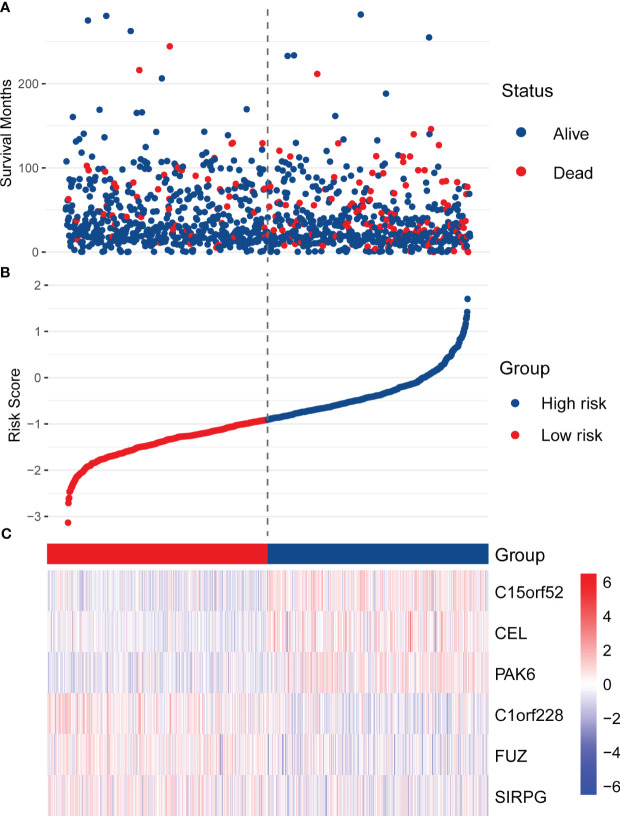
Distribution of RS, survival status, and the expression of six hub genes in the training set. **(A)** The scatter plot shows the distribution of patients’ survival status in the high-risk and low-risk subgroups. **(B)** The distribution of RS in the patients of the training set. **(C)** The heatmap shows the expression of the six hub genes in high-risk and low-risk patients.

The RS of each patient was calculated with the following formula: RS = (0.204 × Exp*_C15orf52_*) + (-0.253 × Exp*_C1orf228_*) + (0.170 × Exp*_CEL_*) + (-0.220 × Exp*_FUZ_*) + (0.302 × Exp*_PAK6_*) + (-0.108 × Exp*_SIRPG_*). With the median as the cut-off value, 1078 patients in the training set were divided into two subgroups, high-risk (n = 539) and low-risk (n = 539). As shown in **Figure 2A**, the BC patients in the high-risk subgroup have higher mortality. The distribution of RS was described in **Figure 2B**, ranging from -3.14 to 1.70, and RS’s median (lower quartile, upper quartile) was -0.91 (-1.34, -0.43). **Figure 2C** presents the expression of 6 hub genes in patients. Since higher expression of *C1orf228*, *FUZ*, and *SIRPG* was associated with better survival of BC patients in the training set, these three genes had a lower expression in the high-risk subgroup than the low-risk subgroup.

**Figure 3:**
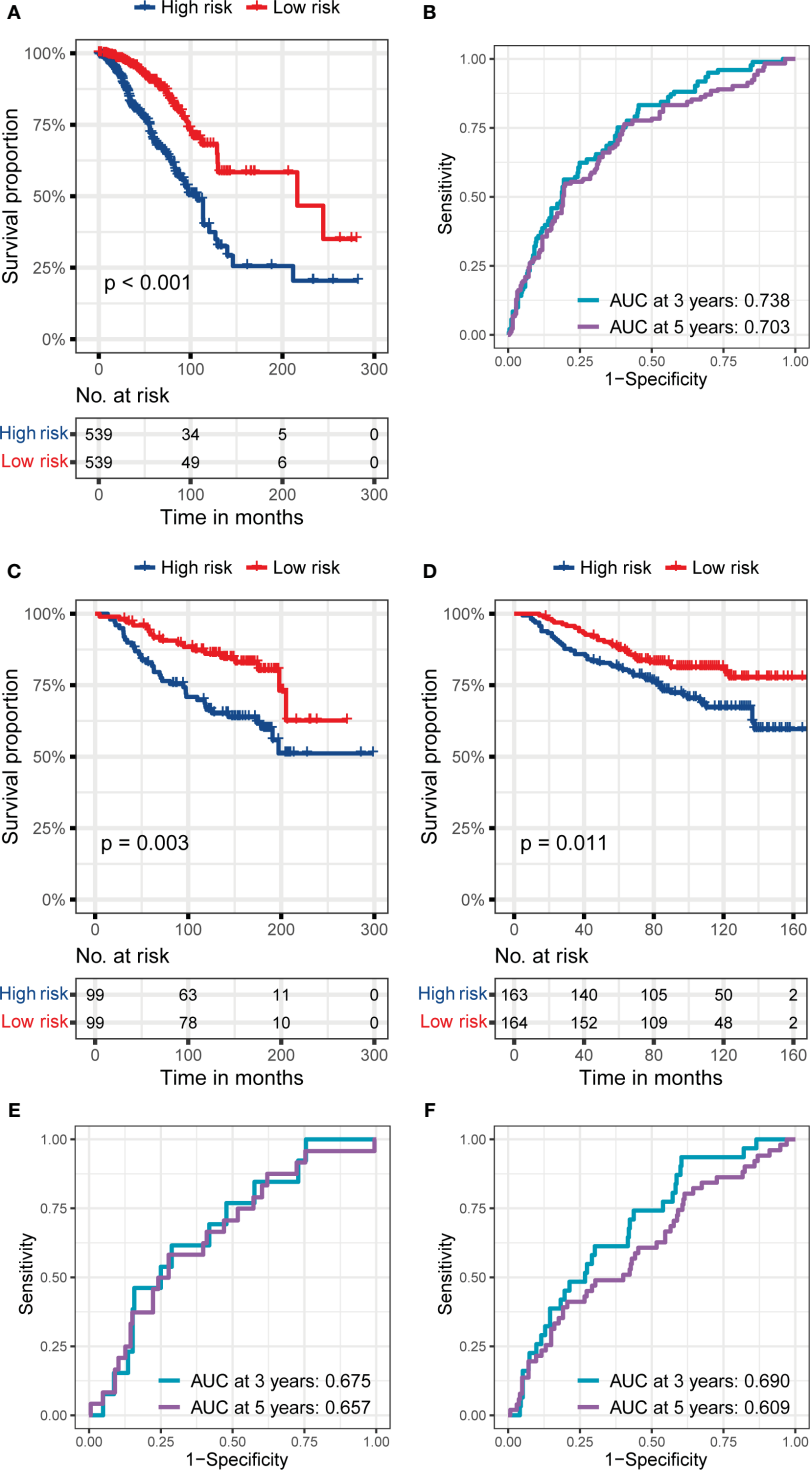
The predictive performance of the six genes RS signature. **(A, B)** K-M analysis and survival curve show significant differences in the survival between high-risk and low-risk subgroups, and ROC curve shows the prognostic value of RS for predicting the 3- and 5-years cut-off OS in the training set (TCGA dataset). **(C, D)** K-M analyses and survival curves show significant differences in the survival between high-risk and low-risk subgroups GSE7390 **(C)** and GSE20685 **(D)**. **(E, F)** ROC curves show the prognostic value of RS for predicting the 3- and 5-years cut-off OS in GSE7390 **(E)** and GSE20685 **(F)**.

Due to the prolonged survival time of BC, we did not use one year as an essential node for survival analysis. **Figures 3A, B** show the RS signature’s performance in predicting patients’ prognosis in the training set. The results indicated that the patients in the low-risk subgroup had a significantly better prognosis than those in the high-risk subgroup (p< 0.001, **Figure 3A**), and the AUC reached 0.738 and 0.701 at 3 and 5 years, respectively (**Figure 3B**).

In the validation sets, the RS signature still had a good performance in predicting the prognosis of BC patients. As presented in **Figures 3C, D**, the BC patients in the low-risk subgroup still had a significantly better prognosis than those in the high-risk subgroup in validation set 1 (p = 0.003, **Figure 3C**), and validation set 2 (p = 0.011, **Figure 3D**). The AUC reached 0.675 at 3 years and 0.657 at 5 years in validation set 1 (**Figure 3E**), while it reached 0.690 at 3 years and 0.609 at 5 years in validation set 2 (**Figure 3F**), proving the prognostic value of RS signature.

### 3.3 The comparison of RS signature with other clinical indicators

**Figure 4:**
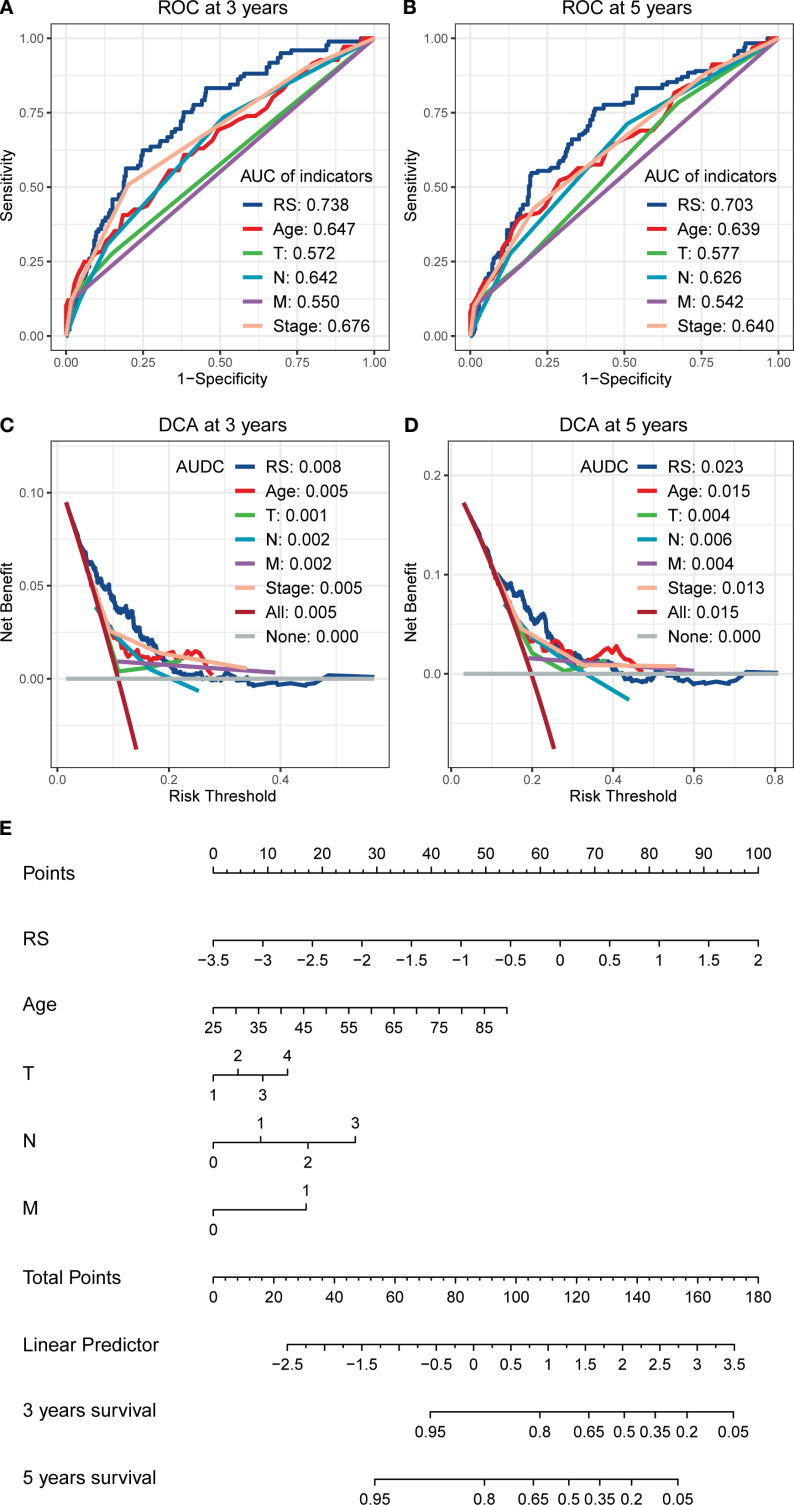
The comparison of RS with other clinical indicators in predicting the prognosis of BC patients. **(A, B)** The ROCs show that the RS has a larger AUC than other clinical indicators, whether in predicting the 3 years cut-off OS **(A)** or 5 years cut-off OS **(B)**. **(C, D)** The DCAs show that the RS has a larger AUDCs than other clinical indicators, whether at 3 years **(C)** or 5 years **(D)**, indicating the value of RS in clinical decision making. **(E)** Nomogram shows the performance of age, T, N, M, and RS in predicting the prognosis of BC patients in the multivariate cox regression analysis.

In the multivariate cox regression, RS showed a more vital predictive ability than other clinical indicators, including age, T, N, M, and TNM stage in the training set. When comparing AUC, RS still had a better performance than other clinical indicators, including age, T, N, M, and TNM stage, whether at 3 years (**Figure 4A**) or 5 years (**Figure 4B**). In DCA, the AUDCs of RS reached 0.008 at 3 years (**Figure 4C**) and 0.023 at 5 years (**Figure 4D**), which were also much higher than other clinical indicators, indicating the high value of RS signature in clinical decision-making. The Nomogram containing age, T, N, M, and RS is presented in **Figure 4E**. It can be seen from the **Figure 4E** that the contribution of RS to the total score is the largest, indicating that RS is more predictive of the patient’s prognostic situation compared to other traditional clinical factors, including age and TNM staging.

### 3.4 Favorable predictive performance of RS signature

**Figure 5:**
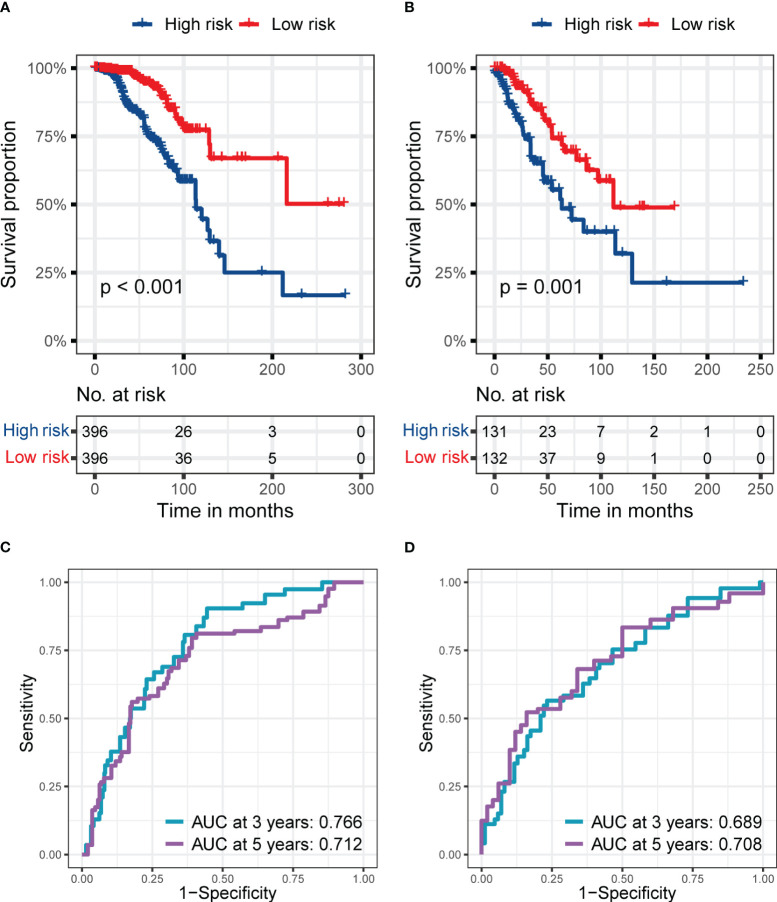
The predictive performance of the six genes RS signature in different stages of patients from the training set. **(A, B)** K-M analyses and survival curves show significant differences in survival between high-risk and low-risk subgroups, whether in patients at stages I and II **(A)** or III and IV **(B)**. **(C, D)** ROC curves show the prognostic value of RS for predicting the 3- and 5-years cut-off OS, whether in patients at stage I and II **(C)** or III and IV **(D)**.

**Figure 5** shows the predictive performance of RS signature in BC patients with different TNM stages in the training set. The RS signature had an excellent predictive performance for OS whether patients were in stages I and II (p< 0.001, **Figure 5A**) or stages III or IV (p = 0.001, **Figure 5B**). The AUC reached 0.766 at 3 years and 0.712 at 5 years in patients with stage I or II (**Figure 5C**), while it reached 0.689 at 3 years and 0.708 at 5 years in patients with stage III or IV (**Figure 5D**).

In **Table 1**, we summarized vital metrics representing the prognostic value of RS signature in ER, PR, HER2 negative or positive patients. Significant p-values in KM analyses between patients in high-risk and low-risk subgroups and large AUC values indicated the good predictive ability of RS signature in BC patients with ER, PR, and HER2 negative or positive. The complete KM and ROC curves are presented in **Figure S2**.

**Table 1:** The predictive performance of the six genes RS signature in patients with different ER, PR, and *HER2* statuses from the training set.

Biomarkers	Status	*p* for KM	AUC	
			3 years	5 years
ER	negative	0.002	0.675	0.736
	positive	<0.001	0.751	0.657
PR	negative	<0.001	0.714	0.738
	positive	0.033	0.708	0.620
*HER2*	negative	<0.001	0.711	0.707
	positive	0.005	0.795	0.716

**Figure 6:**
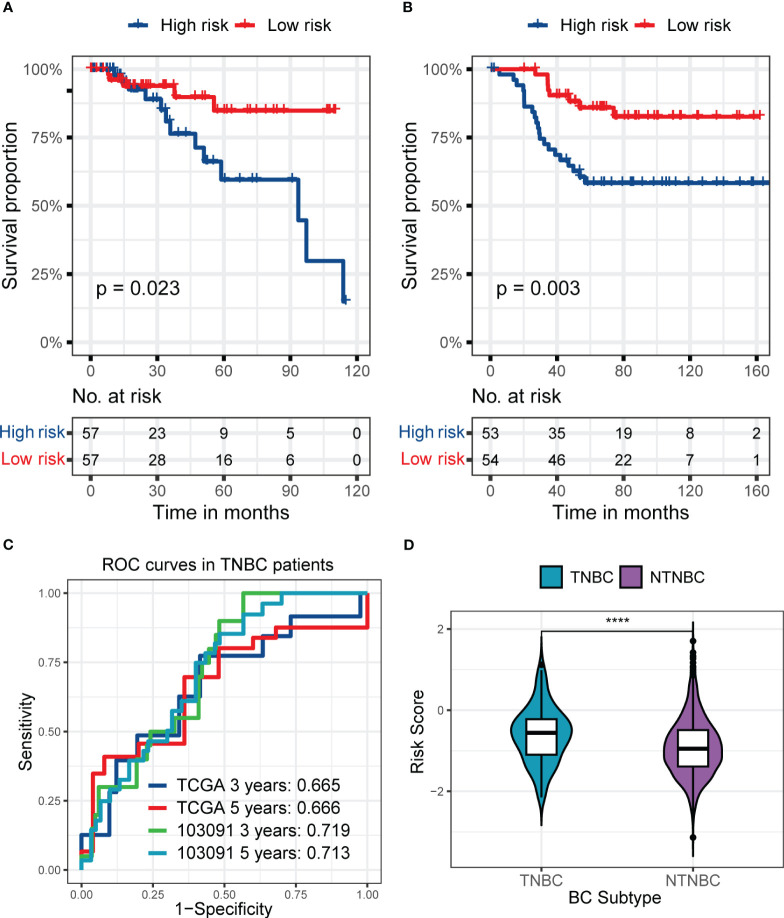
The predictive performance of the six genes RS signature in TNBC patients from the training set or GSE103091 and the distribution of RS in TNBC and NTNBC patients in the training set. **(A, B)** KM analyses and survival curves show significant differences in the survival between high-risk and low-risk subgroups, whether in patients from the training set **(A)** or the GSE103091 **(B)**. **(C)** ROC curves show the prognostic value of RS for predicting the 3- and 5-years cut-off OS, whether in patients from the training set or the GSE103091. **(D)** The Violin plot shows that the TNBC patients had a higher RS than NTNBC patients. (****p ≤ 0.0001).

Furthermore, the prognostic value of RS signature in TNBC patients was examined (**Figure 6**). In the training set, TNBC patients in the high-risk subgroup had worse prognostic performance than the low-risk subgroup (p = 0.023, **Figure 6A**), and the AUC reached 0.665 and 0.666 at 3 and 5 years (**Figure 6C**), respectively. Interestingly, the RS signature had a better performance in the external validation set, GSE103091. With a worse prognosis of patients in the high-risk subgroup (p = 0.003, **Figure 6B**) and the AUC reached 0.719 at 3 years and 0.713 at 5 years (**Figure 6C**), it revealed the prognostic value of RS signature in TNBC patients. Meanwhile, the RS of TNBC patients was significantly higher than that of NTNBC patients (**Figure 6D**), showing the predict value of the RS signature.

### 3.5 Single-cell analysis in TNBC patients

**Figure S3** demonstrates the cluster results of all cells of the nine TNBC samples. Using the median RS as the cut-off, four (containing 12077 cells) of the nine TNBC patients were classified as high-risk and five (containing 23412 cells) were classified as low-risk. We identified a total of 8 major cell classes in TNBC patients (**Figure S3A**), but interestingly, one of these classes (mesenchymal stem cells, MSC) was only found in patients at low risk (**Figures S3B, C**).

**Figure 7:**
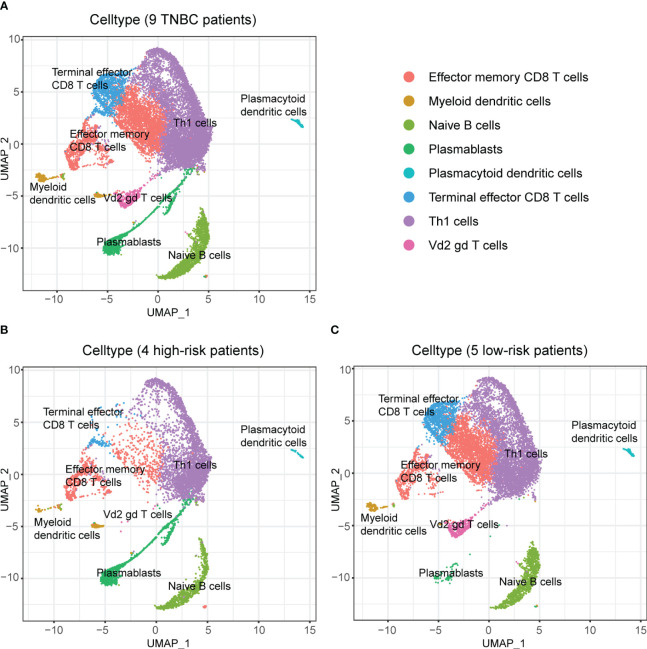
Composition of lymphocytes in nine TNBC patients and their RS subgroups. **(A)** Composition of lymphocytes in nine TNBC patients. **(B)** Composition of lymphocytes in patients with high RS. Some subpopulations of T cells in these patients had low numbers. **(C)** Composition of lymphocytes in patients with low RS. An abundance of multiple subpopulations of T cells was found in these patients.

Afterward, we performed subpopulation analysis for lymphocytes, including B and T cells (**Figure 7**). **Figure 7A** showed the clustering results of lymphocytes in the tumors of the nine TNBC patients, and we observed a high proportion of T cells among the tumor-infiltrating lymphocytes in these nine TNBC patients. However, when further localized these cells to patients with different RS, we were surprised to find a decrease in multiple subtypes of T cells in the four high-risk patients (**Figures 7B, C**).

**Figure 8:**
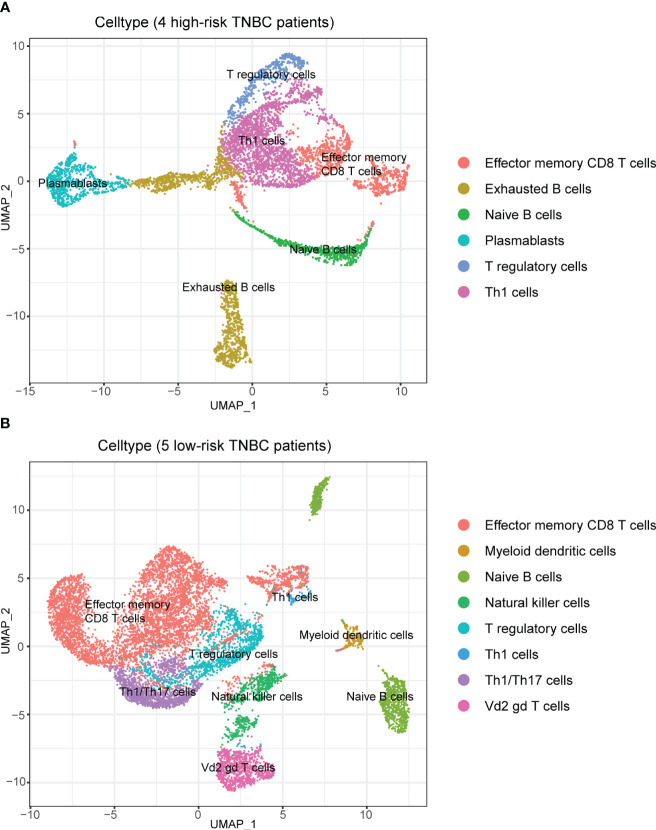
Composition of lymphocytes in patients with different RS when analyzed separately. **(A)** Composition of lymphocytes in patients with high RS. There was a high percentage of B cells among lymphocytes. **(B)** Composition of lymphocytes in patients with low RS. There was an absolute predominance of T cells among lymphocytes.

To avoid the possible influence of differences in overall cell numbers on the results, we further examined the lymphocyte infiltration in the tumor samples of TNBC patients with different RS separately. The results demonstrated that the proportion of all kinds of T cells among lymphocytes decreased in the four patients at high risk than those in the five patients at low risk (**Figures 8A, B**), which indicated the good discrimination ability of the RS signature developed in our study.

At the same time, we also analyzed the expression of hub genes that consisted of the RS signature in different cells (**Figure S4**). The results showed that some of the genes could not be clustered due to the low expression baseline, but it could be observed that three genes, C1orf228, FUZ and SIRPG, showed a tendency to be concentrated in specific T cell subpopulations.

### 3.6 Immune checkpoint analysis in TNBC patients

Compared to low-risk TNBC patients, TNBC patients in the high-risk subgroup showed a lower expression of most immune checkpoint genes, whether in the training set or GSE103091. As presented in **Figures S5A, S5B**, after multiple-testing correction, TNBC patients in the high-risk subgroup showed a lower expression of *BTLA*, *CD200R1*, *CD27*, *CD28*, *CD40*, *CD40LG*, *CTLA4*, *HLA-DRB1*, *IL2RB*, *LAG3*, P*DCD1*, *TIGIT*, and *TNFRSF14* both in two datasets.

### 3.7 Drug sensitivity of TNBC patients with different RS signatures

**Figure 9:**
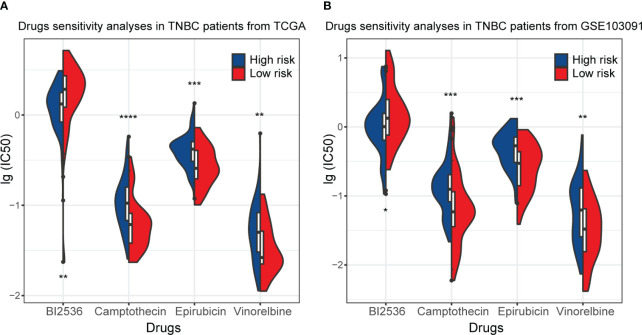
Drug sensitivity of TNBC patients in the training set (TCGA) and GSE103091. **(A)** The distribution of IC50 of BI2536, Camptothecin, Epirubicin, and Vinnorelbine in TNBC patients from the training set. **(B)** The distribution of IC50 of BI2536, Camptothecin, Epirubicin, and Vinnorelbine in TNBC patients from GSE103091. (*p ≤ 0.05, **p ≤ 0.01, ***p ≤ 0.001, ****p ≤ 0.0001).

In this part of the analysis, the sensitivity of 198 drugs that may have efficacy in breast cancer was examined in the training set and GSE103091. Among them, the sensitivity of 82 drugs was statistically different between the high-risk and low-risk subgroups and showed the same trend in the training set and GSE103091. The full results are shown in **Tables S1**, **S2**. Of the 82 kinds of drugs, only one, BI2536, demonstrated a smaller IC50 (which mean a higher drug sensitivity) in the high-risk subgroup in the training set and external validation set GSE103091, while all other drugs had lower sensitivity in the high-risk subgroup (**Figure 9**). These results demonstrated that the patients in the high-risk subgroup distinguished by our RS signature were resistant to many common therapeutic agents, which might provide a basis for more individualized treatment regimens.

## 4 Discussion

In this study, we established an RS signature containing six genes, including *C15orf52*, *C1orf228*, *CEL*, *FUZ*, *PAK6*, and *SIRPG*, which could predict the prognosis of BC patients well and more robust than traditional clinical indicators. In BC patients with different disease stages and molecular subtypes, the RS signature still showed good predictive power, which might benefit BC patients’ treatment and prognosis.

Using multi-omics data could utilize genetic information from multiple dimensions and make more extensive use of data. Our RS signature showed better prognostic effectiveness than previous studies that attempted to find potential biomarkers of the prognosis in BC patients based on single-omics data. In a recent study, Tian and colleagues identified a five-miRNA signature from the TCGA database (30). Their signature performed well in the training set, but the AUC only reached 0.679 even in the internal validation set, and their signature was lack of validation in external datasets. In another study, the authors developed a ten-lncRNA signature based on the data from TCGA-BRCA (31). In entire TCGA-BRCA patients, their AUC reached 0.741 at three years. Although it was comparable to ours, we achieved the same goal of predicting the prognosis of patients with fewer genes. While our RS signature showed a good predictive ability in independent validation cohorts and stratification analyses, these performances could benefit on the prognosis and treatment of BC patients.

Only a few studies have attempted to explore biomarkers of the prognosis of BC patients based on multi-omics data. In a recent article, Fan and colleagues identified 15 genes associated with the prognosis of BC based on multi-omics data from TCGA and molecular taxonomy of the breast cancer international consortium (METABRIC) database (32). However, they did not combine these genes into a signature and did not examine the performance of these genes in different subtypes or stages of BC patients. In another very recent article, the authors identified a five genes prognosis signature in TNBC patients (33). However, its results lacked validation in external datasets compared with this present study. In general, the six genes found and the RS signature constructed in this study showed great value in predicting the prognosis of BC patients in various subtypes. It could accurately predict the prognosis of patients with fewer genes, making it easier to apply to practical work.

The six hub genes that make up the RS signature have all been confirmed to be associated with tumors. *C15orf52*, also known as *CCDC9B*, has been identified as a hub gene of pancreatic ductal adenocarcinoma development in the weighted gene co-expression network analysis (34). *C1orf228*, also called *ARMH1*, is a signature gene in oral squamous cell carcinoma based on random forest methods (35). *CEL*, whose full name is carboxyl ester lipase, has been studied for a long time. In a recent study based on the TCGA database, the authors found that *CEL* could be an independent prognostic factor of BC (36). However, the AUC was poor, indicating that *CEL* expression could not independently distinguish the prognosis of patients (36). It has been shown that the CEL gene and is one of the risk factors for pancreatitis and that pancreatitis patients have a high probability of developing pancreatic cancer, while a direct relationship between the CEL gene and pancreatic cancer cannot be completely ruled out (37). *FUZ* is a member of planar cell polarity genes. The study has revealed the correlation between the expression of planar cell polarity genes and tumor cell viability (38). The expression of *FUZ* itself has also been associated with the prognosis of many cancers in a recent pan-caner study (39). *FUZ* has been found to play a role in promoting tumor growth in patients with non-small cell lung cancer that has metastasized, and has the potential to play an important role in the treatment of patients with small cell lung cancer (40). *PAK6* is a member of the p21-activated kinases (PAKs) family. The overexpression of PAKs was considered to have oncogenic signaling effects and has been found in various tumors (41). One study found that *PAK6* affects the efficacy of chemotherapy in gastric cancer patients and also modulates tumor resistance (42)*. SIRPG* has been found to affect many other diseases, but its research is fewer in tumors. Recent studies found that *SIRPG* could become a potential biomarker for endometrial carcinoma and head and neck squamous cell carcinoma (43, 44) and might promote the immune escape of tumor cells in lung cancer (45). In a study by Wang et al. it was shown that there was a significant relationship between PD-1 and overall survival time in patients with high-grade plasma ovarian cancer, as well as a highly correlated relationship between *SIRPG* and PD-1, thus linking *SIRPG* to the prognosis of the patients (46). Though these six hub genes have been reported to be associated with tumors in previous studies, the function of most of them is still unclear and requires further research to clarify.

Although BC was not initially considered an immunogenic cancer type, more evidence has supported that antitumor immunity had an important role, especially in subtypes like TNBC or *HER2*-positive BC (47). We also explored the performance of our RS signature using single-cell data in TNBC patients.

It was observed that many immune cells infiltrated in TNBC tumor tissue, which partially validated that TNBC could be a kind of immunogenic cancer. Interestingly, the distribution of cells in TNBC patients with different RS was different in the single-cell data analysis. In patients with higher RS, we did not find any cells belonging to the MSC subpopulation. MSC was thought to play the opposite role in tumor development. On the one hand, it could inhibit AKT and Wnt signaling pathways, as well as angiogenesis, to suppress tumor growth (48–52). On the other hand, it has been found to inhibit immunity and tumor cell apoptosis, thereby favoring tumor growth and metastasis (53–56). Currently, studies have attempted to deliver and express various anti-tumor drugs through MSC, showing potential and leading to a new therapeutic approach (57–60). Therefore, the discovery of MSC in the tumor tissue of part of TNBC patients may provide beneficial information for clinical treatment. Meanwhile, this finding that MSC was only found in TNBC patients with lower RS indicates a different cellular profile among the patients distinguished by our RS signature, which also provides good support for individualized therapeutic regimens.

Among the subpopulation analysis targeting lymphocytes in the tumor samples of TNBC patients with different RS separately, we observed that the proportion and number of T cells were decreased in the samples from high-risk patients compared with low-risk patients. T lymphocytes were considered to dominate most adaptive immune responses against tumors (61). The reduction of T cells in the cellular component of high-risk patients may reflect their poor tumor immune, which could partially explain their worse prognosis and the decrease of many immune checkpoint expressions observed in the previous immune checkpoint analysis.

In any case, as validated by single-cell data, the cellular composition of the tumor tissue differed considerably between patients distinguished by our RS signature, which would facilitate the development of more personalized treatment plans.

In the drug sensitivity analysis, we found that most drugs had a lower sensitivity in the high-risk group except for BI2536, which has a higher sensitivity in the high-risk group. BI2536 is a kind of inhibitor of polo-like kinases (62). Current studies have identified the antitumor effects of BI2536 in various cancers (63–65), but relatively little research has been done in BC. A few studies have shown that combining BI2536 with other drugs in BC may enhance the latter’s antitumor activity (66); however, few studies have been conducted for BI2536 alone. Our study found that the sensitivity of tumor cells to BI2536 in the high-risk group of TNBC patients was very different from that of other drugs, revealing that BI2536 may have a unique effect in patients with TNBC and facilitate the development of a more individualized treatment plan, suggesting the implication of further studies.

The RS signature consisted of six genes established in the present study that performed well. However, if the following limitations were overcome, it might have a more outstanding contribution to the prognosis of BC patients. First, limited data accessibility made obtaining disease-free survival (DFS) in all data sets difficult. So, OS was adopted as the primary outcome in the survival analysis, which might cause bias and reduce the accuracy of prognosis judgment for BC patients. Second, in the survival analysis, the risk status of patients was dichotomized using a cohort-specific median of risk score, which requires future research in a larger cohort to identify the cut-off value.

In conclusion, we established a six genes RS signature based on multi-omics data, which had good performance in predicting the prognosis of BC patients in different disease stages or subtypes. It could contribute to a more personalized treatment, which might benefit the outcome of BC patients.

## Data availability statement

The data used to support the findings of this study are available at UCSC (https://xenabrowser.net/datapages/) and GEO (http://www.ncbi.nlm.nih.gov/geo/) databases.

## Author contributions

Conceptualization, ZX and DL; Data curation, LX, MY and XX; Formal analysis, YH and ZM; Funding acquisition, XW and JG; Investigation, LX, MY and XX; Methodology, HJ, YL, JLS and JRS; Project administration, XW and JG; Resources, TW, HZ and XC; Software, HJ, YL, JLS and JRS; Validation, TW, HZ and XC; Visualization, YH, ZM, HJ, YL, JLS and JRS; Writing – original draft, ZX, DL, YH and ZM; Writing – review & editing, XW and JG. All authors contributed to the article and approved the submitted version.
