# Supplementary material for: Construction of a prognostic 6-gene signature for breast cancer based on multi-omics and single-cell data
Source: Front Oncol. 2023 Nov 21;13:1186858. doi: 10.3389/fonc.2023.1186858 (PMC10698552; doi:10.3389/fonc.2023.1186858)
Supplement: Supplementary file 3 [file DataSheet_1.docx]

Supplementary Material

Construction of a prognostic 6-gene signature for breast cancer based on multi-omics and single-cell data

Zeyu Xing^†^, Dongcai Lin^†^, Yuting Hong^†^, Zihuan Ma^†^, Hongnan Jiang, Ye Lu, Jiale Sun, Jiarui Song, Li Xie, Man Yang, Xintong Xie, Tianyu Wang, Hong Zhou, Xiaoqi Chen, Beifang Niu^*^, Yipeng Wang^*^, Jidong Gao^*^

^†^These authors contributed equally to this work.

**^*^Correspondence:** Beifang Niu: [beifangniu@chosenmedtech.com](mailto:beifangniu@chosenmedtech.com), [bniu@sccas.cn](mailto:bniu@sccas.cn); Yipeng Wang: [yidoctor99@126.com](mailto:yidoctor99@126.com); Jidong Gao: [ab168@cicams.ac.cn](mailto:ab168@cicams.ac.cn)

# Supplementary Figures and Tables

## Supplementary Figures


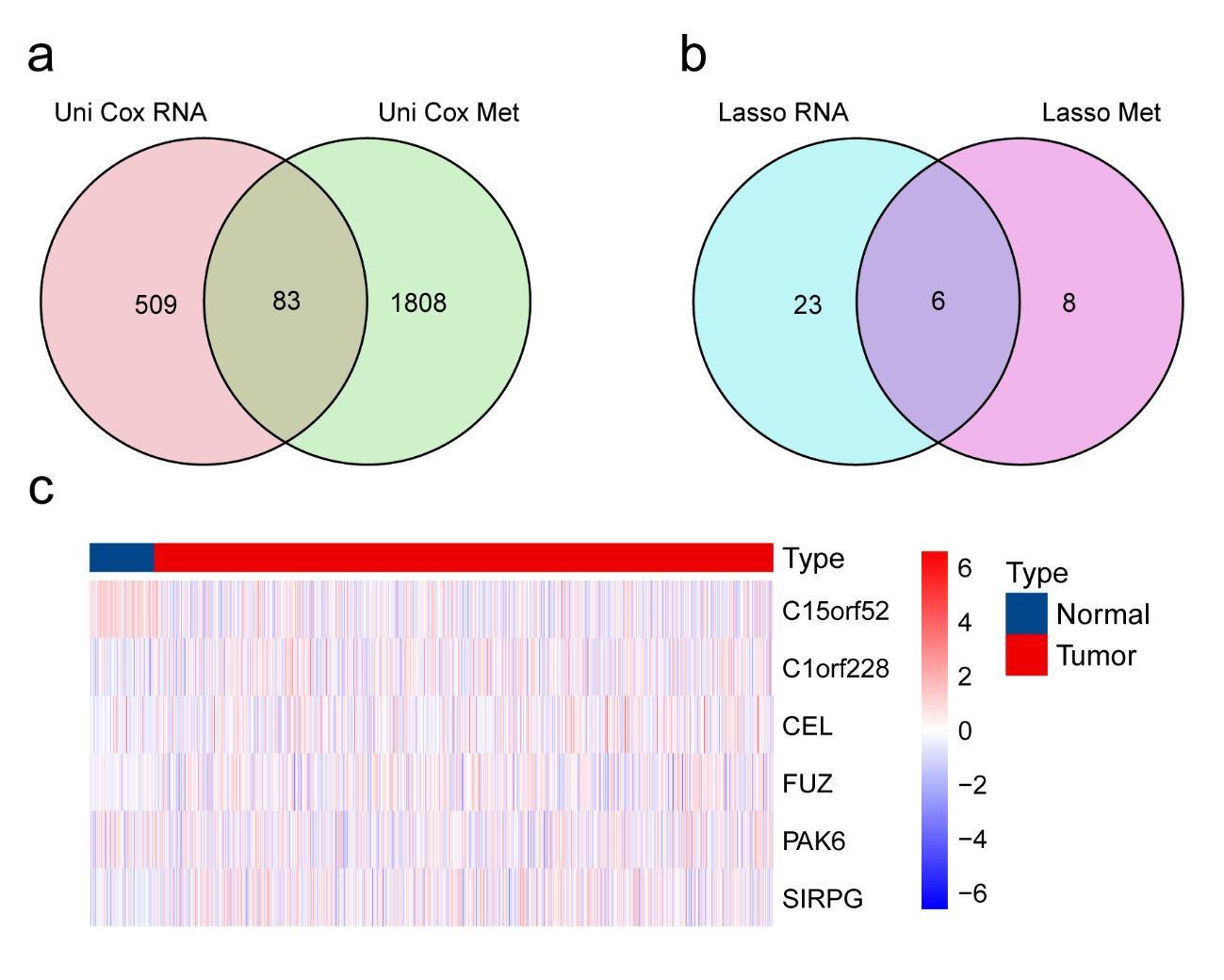


**Figure S1.** Gene screening and expression in different tissues in the training set. **(a)** The Veen diagram shows 83 overlapped genes in the univariate cox analysis. **(b)** The Veen diagram shows six overlap genes in the Lasso analysis. **(c)** The heatmap shows the expression of the six hub genes in normal and tumor tissue.


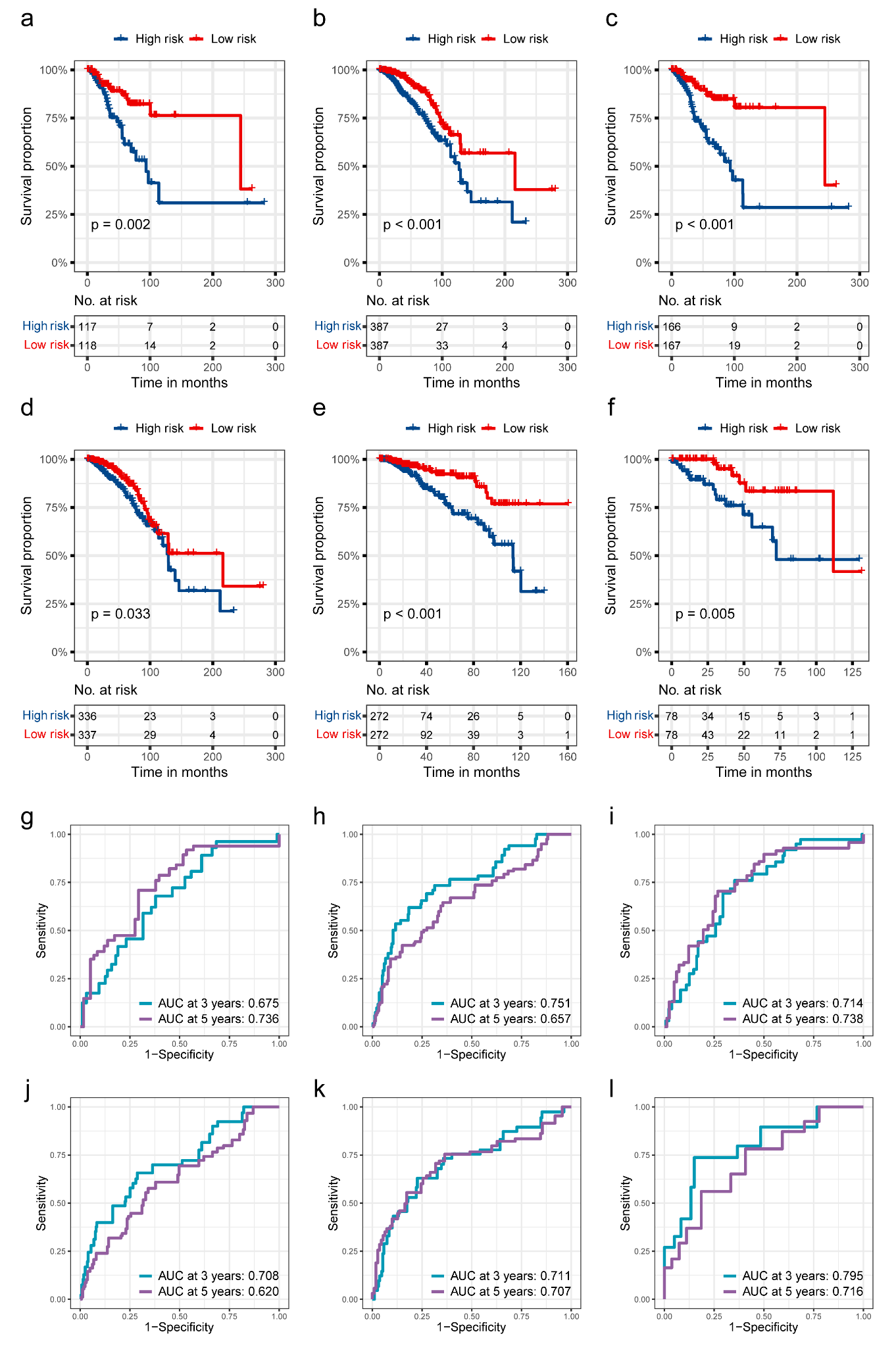


**Figure S2.** The survival and ROC curves of the six genes RS signature in patients with different ER, PR, and HER2 statuses from the training set. **(a-f)** K-M analyses and survival curves show that there are significant differences in the survival between high-risk and low-risk subgroups whether in patients with ER negative **(a)** and positive **(b)**, PR negative **(c)** and positive **(d)**, or HER2 negative **(e)** and positive **(f)**. **(g-l)** ROC curves show the prognostic value of RS for predicting the 3- and 5-years cut-off OS, whether in patients with ER negative **(g)** and positive **(h)**, PR negative **(i)** and positive **(j)**, or HER2 negative **(k)** and positive **(l)**.


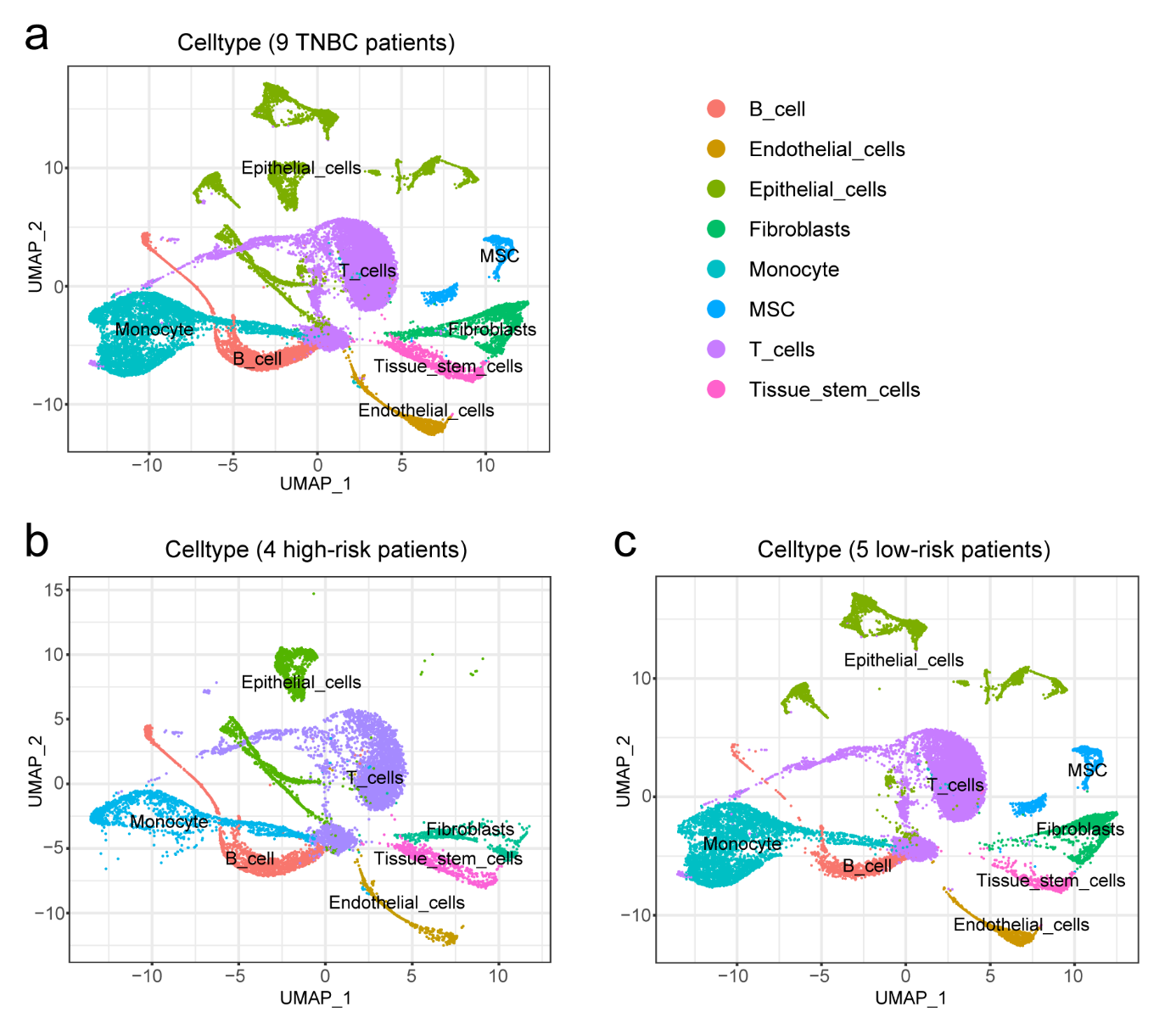


**Figure S3.** Composition of cells in nine TNBC patients **(a)** and patients with different RS subgroups in the nine patients **(b-c)**.


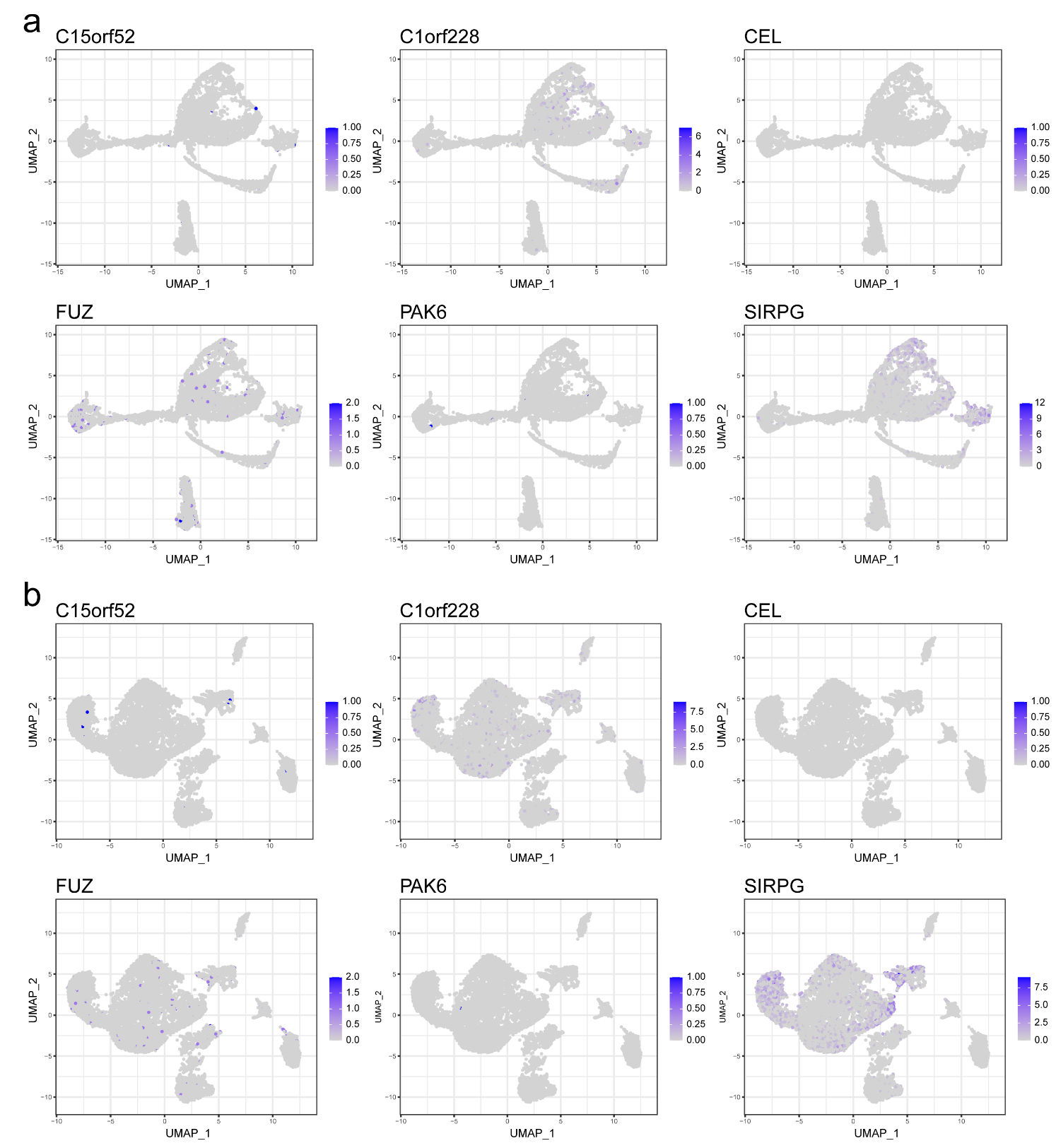


**Figure S4.** Expression of six hub genes in lymphocytes from patients with high RS **(a)** and low RS **(b)**.


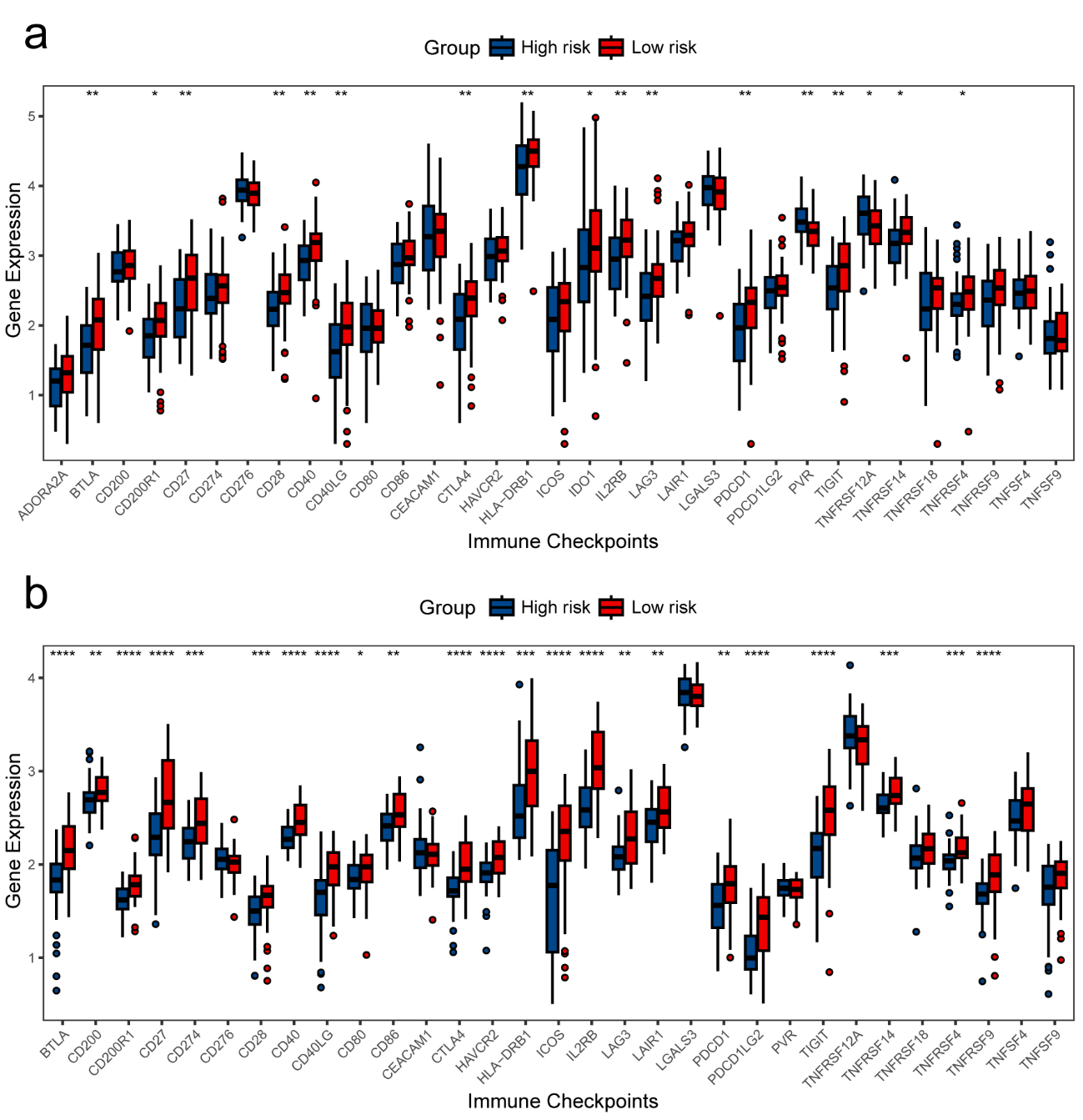


**Figure S5.** The distribution of immune checkpoints expression in TNBC patients from the TCGA-BRCA and GSE103091. **(a)** Comparison of immune checkpoints expression between high-risk and low-risk groups in TNBC patients from TCGA-BRCA (training set). **(b)** Comparison of immune checkpoints expression between high-risk and low-risk groups in TNBC patients from GSE103091. (*: adjusted *p* ≤ 0.05, **: adjusted *p* ≤ 0.01, ***: adjusted *p* ≤ 0.001, ****: adjusted *p* ≤ 0.0001)

## Supplementary Tables

**Table S1 (supplied as separate file).** Results of drug sensitivity analysis in TCGA-BRCA dataset.

**Table S2 (supplied as separate file).** Results of drug sensitivity analysis in GSE103091.
